# Extreme low temperature tolerance in woody plants

**DOI:** 10.3389/fpls.2015.00884

**Published:** 2015-10-19

**Authors:** G. Richard Strimbeck, Paul G. Schaberg, Carl G. Fossdal, Wolfgang P. Schröder, Trygve D. Kjellsen

**Affiliations:** ^1^Department of Biology, Norwegian University of Science and TechnologyTrondheim, Norway; ^2^Northern Research Station, United States Department of Agriculture Forest Service, BurlingtonVT, USA; ^3^Norwegian Forest and Landscape InstituteÅs, Norway; ^4^Department of Chemistry, Umeå UniversityUmeå, Sweden

**Keywords:** cold, frost, tolerance, hardiness, acclimation, hardening, biochemistry, vitirification

## Abstract

Woody plants in boreal to arctic environments and high mountains survive prolonged exposure to temperatures below -40°C and minimum temperatures below -60°C, and laboratory tests show that many of these species can also survive immersion in liquid nitrogen at -196°C. Studies of biochemical changes that occur during acclimation, including recent proteomic and metabolomic studies, have identified changes in carbohydrate and compatible solute concentrations, membrane lipid composition, and proteins, notably dehydrins, that may have important roles in survival at extreme low temperature (ELT). Consideration of the biophysical mechanisms of membrane stress and strain lead to the following hypotheses for cellular and molecular mechanisms of survival at ELT: (1) Changes in lipid composition stabilize membranes at temperatures above the lipid phase transition temperature (-20 to -30°C), preventing phase changes that result in irreversible injury. (2) High concentrations of oligosaccharides promote vitrification or high viscosity in the cytoplasm in freeze-dehydrated cells, which would prevent deleterious interactions between membranes. (3) Dehydrins bind membranes and further promote vitrification or act stearically to prevent membrane–membrane interactions.

## Introduction

Much of the more than 100-year-old body of literature on low temperature (LT) tolerance in plants is focused on herbaceous crop species such as cereal grasses, potato, alfalfa and, more recently, *Arabidopsis*, with more limited work on woody plants including fruit and ornamental species and some important forest species (e.g., Sakai and Larcher, 1987; Gusta et al., 2009; Wisniewski and Gusta, 2014). These species may be subject to stress, injury, or death due to LT stress when grown under marginal or changing temperature regimes. Therefore, an understanding of the genetics and mechanisms of LT tolerance may ultimately lead to improved stress resistance and productivity through breeding or genetic engineering. These studies have yielded considerable insight into the molecular and biophysical mechanisms and functional genomics of tolerance of temperatures as low as -50°C. However, relatively little attention has been paid to plants that naturally survive some of the lowest temperatures on Earth, which are the subject of this review. We will show that extreme low temperature (ELT) tolerance is qualitatively different from more levels that are moderate and likely involves unique biochemical and biophysical survival startegies.

In the taiga forest regions of Siberia and Canada, temperatures range from record lows of around -64°C to record highs of 36°C, thereby spanning a full 100°C. The mean monthly temperatures for December, January, and February are all around -40°C. Many of the plants and animals in this extreme environment overwinter under the protection of snow or in the soil. In contrast, evergreen pine (*Pinus*), spruce (*Picea*), and fir (*Abies*) species, along with deciduous larch (*Larix*) and a few angiosperm tree and shrub species, remain exposed above the snow and survive the extreme cold, variable light, dry conditions, and high winds of the regions’ winters. Similarly, plants in arctic regions and habitats where there is little snow may be exposed to ELT and other stresses for months at time.

Exposed plants in these harsh environments likely employ mechanisms of LT tolerance that go well beyond those of the well-studied species. An understanding of how these plants survive can contribute to crop improvement and technologies for dry and frozen preservation of foods, drugs, and other biological materials (e.g., Langis and Steponkus, 1990; Tada et al., 1990; Siow et al., 2007). Because the phenology of dormancy and LT tolerance may be affected by global warming (Kramer et al., 2000), which is generally expected to be greatest in winter and at higher latitudes, results of this work may also be applied in understanding and predicting the implications of global warming for individual tree species and forest ecosystems, especially in boreal regions.

## Exploring the Limits

Minimum survival temperatures vary according to the natural environment, acclimation state, and growth form of the plant and, in many cases, may vary among different organs and tissues within the plant (Larcher, 2003). Tissues of chilling intolerant lowland tropical plants that never experience subfreezing temperatures can be killed at temperatures between 0 and 10°C, while tissues that can survive these temperatures but not freezing temperatures are referred to as chilling tolerant. Plants from regions with episodic or persistent seasonal temperatures below 0°C are usually described using pairs of the words frost, freezing, or cold and tolerant, hardy, or resistant, with the terms tolerance, hardiness, and resistance used to describe the phenomenon of survival at LT. In this review we use the terms LT tolerant and tolerance to cover the full spectrum of hardiness levels, with the following temperature ranges and abbreviations used to categorize plants at the lower end of the range: moderate low temperature (MLT), -20 to -40°C; intermediate low temperature (ILT), -40 to -60°C; and ELT, <-60°C. While the focus is on ELT tolerant plants, comparisons between these three groups will help highlight the special features of ELT tolerance.

**FIGURE 1:**
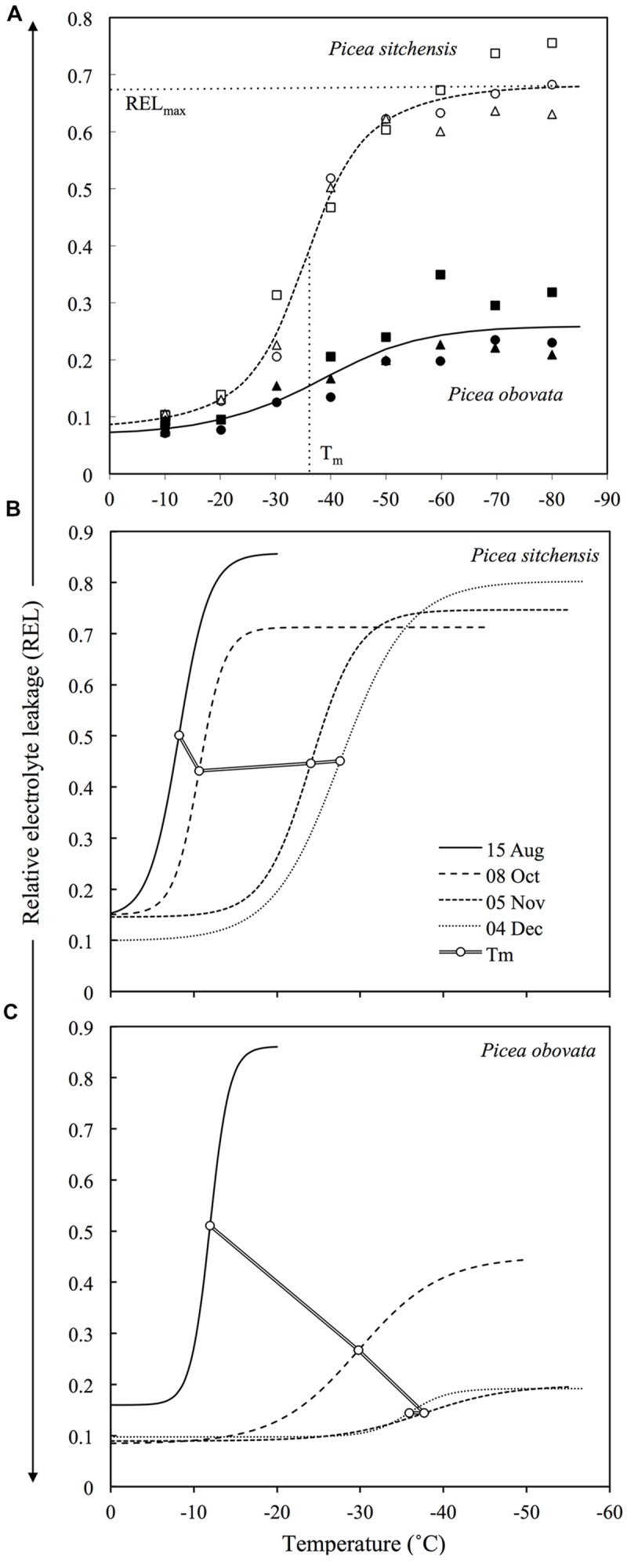
**(A)** Example of REL data and temperature response curves for fully acclimated needles from MLT and ELT tolerant species (*Picea sitchensis* and *Picea obovata*, respectively). Different symbols represent three different trees for each species. Horizontal and vertical dashed lines show location of the parameters REL_max_ and T_m_, respectively for *P. sitchensis* (adapted from Strimbeck et al., 2007). **(B,C)** Changes in temperature response curves and T_m_ during acclimation for *P. sitchensis* **(B)** and *P. obovata* **(C)** (adapted from Strimbeck et al., 2008).

Plant species and varieties can be ranked or categorized by minimum temperatures in their natural ranges or known survival under field conditions, with the hardiness zones defined by US Department of Agriculture often used as a reference (e.g., Bannister and Neuner, 2001). More systematic exploration of LT tolerance requires quantitative estimates of minimum survival temperatures for whole plants or plant tissues. These can be assessed by a wide variety of methods. Typically, whole plants or plant parts are exposed to a range of subfreezing temperatures in a temperature-controlled chamber, although some studies have applied LT treatments to intact plants in the field (e.g., Taschler et al., 2004). LT stress results in various observable or measurable symptoms of injury including death of whole plants, visible necrosis of specific tissues and organs, or less obvious cellular symptoms that can be detected by vital staining, osmotic responsiveness, chlorophyll fluorescence, or measurement of relative electrolyte leakage (REL) in affected tissues. The latter gives a useful measure of injury because a general symptom of cellular injury is a loss of semipermeability of the plasma membrane, which then results in the release of intracellular electrolytes (Dexter et al., 1932; Palta and Li, 1980; Steponkus, 1984). These kinds of measurements are often used to determine a minimum survival temperature or construct temperature response curves and interpolate the temperature resulting in 50% plant or tissue death, LT_50_, (**Figure 1A**). Under natural conditions, trees may be subject to more complex environmental conditions than those imposed in laboratory tests, such as prolonged LT, repeated freezing and thawing, solar warming followed by rapid cooling, or light stress, so that laboratory estimates may not correspond to minimum survival temperatures in the field. In general, laboratory estimates of LT_50_ or minimum survival temperature are somewhat to well below the minimum temperatures encountered in the sampling location or natural range of the species in question. When different methods are directly compared, they often give generally similar estimates of LT_50_ (e.g., Burr et al., 1990), and LT_50_ values based on the same or similar methods can be compared among different tissues and species or track relative changes in LT tolerance over time.

Using these methods, ELT tolerance has been documented in at least 28 angiosperm and 45 gymnosperm species (**Table 1**). Much of this comparative work was done by Sakai (Sakai, 1970, 1983; Sakai and Okada, 1971), including a wide-ranging study of over 70 MLT to ELT tolerant angiosperm and gymnosperm species sampled from North American climate regions ranging from warm temperate to boreal (Sakai and Weiser, 1973). More recently, Strimbeck et al. (2007) compared midwinter LT tolerance parameters in 24 conifer species growing in a common environment at a botanical garden in Trondheim, Norway and Kreyling et al. (2015) compared autumn, winter, and spring LT tolerance in 27 angiosperm and conifer tree species in a botanical garden in Bayreuth, Germany. Both gardens are located in regions with relatively mild winter climates. Despite this, LT tolerance varied according to the climate in the region of origin with Siberian and Canadian species exhibiting full ELT tolerance, indicating that LT tolerance is under strong genetic control.

**Table 1:** Minimum temperatures (°C) for complete survival in tissues of ELT tolerant angiosperm **(A)** and gymnosperm **(B)** tree and shrub species reported in the literature, based on laboratory freezing tests.

(A) Angiopserms				
Species	Buds	Stem/ bark	Source
*Acer saccharum*	-80	-80	f
*Betula nigra*	<-80	<-80	f
*Betula papyrifera*	-15LN	-15LN	f
*Betula pubescens*		-40LN	l,k
*Betula tauschii*		-15LN	e
*Cornus sericea*		-40LN	h
*Fraxinus excelsior*	-50LN		o
*Morus alba*		-30LN	b
*Populus balsamifera*	-15LN	-15LN	f
*Populus sieboldi*		-30LN	b
*Populus tremuloides*	-15LN	-15LN	f, j
*Populus trichocarpa*	<-60	<-60	f
*Quercus macrocarpa*	<-60	<-60	f
*Quercus robur*	-50LN		o
*Quercus rubra*	-50LN		o
*Robinia pseudoacacia*		-70LN	c
*Salix koriyanagi*		-30LN	b
*Salix nigra*	<-80	<-80	f
*Salix sachalinensis*		-15LN	d,e
*Salix scouleriana*	<-60	<-60	f
*Tilia americana*	<-80	<-80	f
*Tilia cordata*	-50LN		o
*Tilia tomentosa*	-50LN		o
*Ulmus americana*	<-80	<-80	f
*Celtis occidentalis*	(-40)	<-80	f
*Fraxinus pennsylvanica*	(-40)	<-70	f
*Juglans nigra*	(-30)	<-80	f
*Populus deltoides*	(-50)	<-80	f

**(B) Gymnosperms**				

**Species**	**Buds**	**Needles/leaves**	**Stem/bark**	**Source**

*Abies balsamea*	-30LN	-30LN	<-80	f,e,n
*Abies sibirica*	-70	<-80		n,g
*Larix dahurica*	-70			g
*Larix decidua*	-50LN			o,e
*Larix laricina*	-15LN		-15LN	f,e,g
*Larix sibirica*	<-120		-70	e,g
*Juniperus communis*	-60	-60		g
*Picea abies*		-50LN		o,n
*Picea engelmanii*	-60	<-70	<-70	f,e,i
*Picea glauca*	<-80	<-80	<-80	f,e,g,n
*Picea mariana*	<-80	<-80	<-80	f,e,g
*Picea obovata*	-70	<-80		n,g
*Picea pungens*	-60	<-80	<-80	f
*Pinus aristata*	-90	-90	-90	e,f
*Pinus banksiana*	-30LN	-30LN	-30LN	f,e,g
*Pinus cembra*	-70	<-80		n,g
*Pinus contorta*	-90	-90	-90	e,f,g
*Pinus koraiensis*	-90	-90	-60LN	e,g,n
*Pinus monticola*	-90	-90	-90	e,f
*Pinus mugo*	-90	-90	-90	e
*Pinus nigra*		-50LN		o
*Pinus parviflora*	-90	-90	-90	e
*Pinus peuce*	-90	-50LN	-90	o,e
*Pinus pumila*	-90	-90	-90	e,g
*Pinus reinosa*	-90	-148	-60LN	f,e,j
*Pinus rigida*	-70	-70	-70	e
*Pinus rostrata*	-90	-90	-90	e
*Pinus strobus*	-50LN	-50LN	-30LN	f,a,e,g
*Pinus sylvestris*	-90	-30LN	-60LN	e,g,m,n,o
*Thuja occidentalis*	-50LN	-50LN	-50LN	f,e
*Tsuga canadensis*	<-60	-70	-60	f,e
*Abies lasiocarpa*	(-40)	<-80	<-80	f
*Abies concolor*	(-40)	<-80	<-80	f,e,g
*Abies holophylla*	(-25)	(-25)	-70	e
*Abies nephrolepis*	(-45)	-70	-70	g
*Abies procera*	(-40)	-70		g
*Abies sachalinensis*	(-45)	-70	-70	f, e
*Abies veitchii*	(-25)	-70	-70	e,g
*Picea abies*	(-35)	<-70	<-70	e
*Picea asperata*	(-45)	-70		g
*Picea glehnii*	(-45)	<-70	<-70	e,g
*Picea jezoensis*	(-45)	-70		g
*Picea omorika*	(-30)	<-70	<-70	e
*Picea rubens*	(-35)	-60	-60	e
*Pseudotsuga menziesii*	(-50)	-70	-80	f

*“LN” indicates survival of liquid nitrogen quenching after slow cooling to the given temperature. “<” indicates survival at the lowest temperature employed in LT testing, so that survival at lower temperatures has not been verified. Parentheses denote minimum survival temperatures above -60, defined as the upper limit of ELT. Reported values give the greatest degree of LT tolerance reported in the literature, with the reference for the table values given first in the Source column and secondary sources listed after. Sources: a, Parker, 1959; b, Sakai, 1960; c, Siminovitch et al., 1968; d, Sakai, 1970; e, Sakai and Okada, 1971; f, Sakai and Weiser, 1973; g, Sakai, 1983; h, Guy et al., 1986; i, Burr et al., 1989; j, Sutinen et al., 1992; k, Rinne et al., 1998; l, Cox and Stushnoff, 2001; m, Repo et al., 2001; n, Strimbeck et al., 2007; o, Kreyling et al., 2015.*

**Table 1** lists numerous cases where stem, bud or needle tissues survive quenching (immersion) in liquid nitrogen (LN_2_) at -196°C after slow cooling to some intermediate temperature. Sakai (1960) was the first to demonstrate and explore this phenomenon. He used regrowth tests to demonstrate that twigs of *Morus, Salix*, and *Populus* species can survive LN_2_ quenching (Sakai, 1960) or even quenching in liquid helium at -269°C (Sakai, 1962b) provided they are first slowly cooled to -30°C. In later work, he found that twigs of some *Salix* and *Populus* species could completely survive quenching from temperatures as high as -15°C (Sakai, 1965). Survival of LN_2_ quenching after precooling to temperatures in the -20 to -40°C range has been confirmed in subsequent work on various ELT tolerant species (**Table 1**). While ELT tolerance can be generally defined as the ability to survive freezing, at least under laboratory conditions, to temperatures below -60°C, these and numerous other studies show that tissues of species from boreal and arctic environments can survive at temperatures approaching absolute zero (-273°C) indicating “absolute” LT tolerance.

The majority of ELT tolerant species originate in boreal interior or cold temperate mountain regions where minimum temperatures fall below -40°C. Where LT_50_ or minimum survival temperatures for buds are > -60°C the species cannot be considered fully ELT tolerant even though other tissues may survive at lower temperatures (**Table 1**). Most of these partially ELT tolerant species originate in somewhat warmer climate regions. In some cases, LT tolerance has been shown to vary within a species’ range or among seed sources within the range (e.g., Sakai and Weiser, 1973), with ELT tolerance found in the colder parts of the range.

## Beyond LT_50_: Interpreting Temperature Response Curves

**Table 1** shows that numerous ELT tolerant species can completely survive at temperatures as low as -80°C or even immersion in LN_2_ at -196°C. In studies employing scoring of whole plant survival or visible injury symptoms as the main response variable, it is not possible to determine LT_50_ if none of the freezing treatments produce at least 50% injury. Similarly, in studies using REL or other relative measures, LT_50_ cannot be determined unless the lowest test temperature completely kills the tissue to give a reference value for 100% injury. This is why most of the studies shown in **Table 1** give minimum temperatures for complete survival, often the minimum temperature that can be achieved by the laboratory freezing system, rather than LT_50_s. In a few cases, we have reinterpreted published freezing response data to estimate minimum survival temperature in order to be consistent.

Electrical conductivity measurements are made by soaking samples in deionized water, sometimes with a low concentration of detergent to improve sample wetting, for a specified amount time, and then measuring the conductivity of the solution with an electrode. REL is generally calculated as the conductivity of a control or freeze-stressed sample to the conductivity of the same sample after it is killed by heat. Although there is considerable variation in the details of the method and subsequent analysis, it remains as one of the most widely used methods of assessing plant LT tolerance due to its convenience and reproducibility. REL can in principle vary from 0 to 1 (or 0–100%) depending on the degree of injury produced by freezing treatment. In practice, REL is generally around 0.1–0.2 in unstressed samples because sample preparation often involves cutting the tissue that damages some cells. It is also usually <1.0 even in freeze-killed samples, most likely because autoclaving releases ions bound in proteins or other cell components. Sigmoid response curves can be fitted using a logistic or similar function (e.g., Anderson et al., 1988), which allows objective estimation of REL at the lower and upper asymptotes (REL_min_ and REL_max_).

**FIGURE 2:**
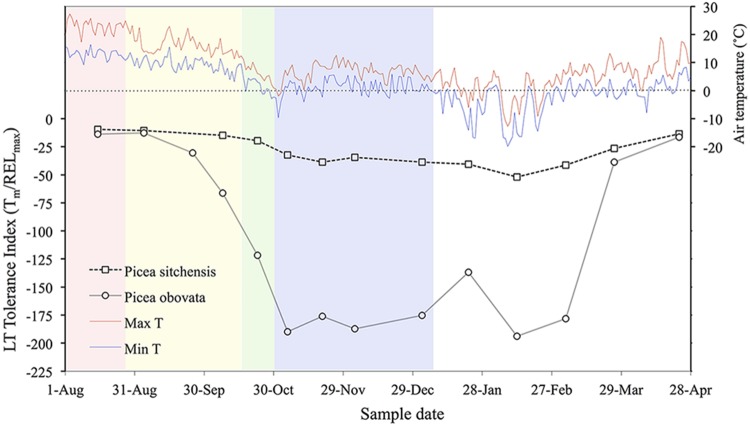
**Daily maximum and minimum temperatures and seasonal acclimation and deacclimation in *Picea sitchensis* (an MLT tolerant species from a temperate oceanic environment) and *P. obovata* (an ELT tolerant Siberian species).** Colored backgrounds indicate acclimation phases in *P. obovata* determined by cluster analysis of metabolomic data: pink, pre-acclimation; yellow, early acclimation; green, late acclimation; blue, fully acclimated (adapted from Strimbeck et al., 2008; Angelcheva et al., 2014).

Freezing treatment of even the most ELT tolerant species gives reproducible sigmoid REL response curves, albeit with a lower amplitude than in more sensitive species (**Figure 1A**). In these curves, REL_max_ indicates the maximum response to stress that can be achieved by slow freezing and provides an important second measurement for comparative assessment of LT tolerance in ELT versus MLT species. In fully acclimated MLT tolerant species, maximum REL values produced by freezing stress (REL_max_) are usually around or above 0.7, while in ELT tolerant species they may range from 0.2 to 0.4 (**Figure 1A**), even after LN_2_ quenching (Strimbeck et al., 2007). The muted increase in REL in the latter group indicates that there is some sub-lethal physiological effect at the cellular level, most likely on the plasma membrane, that results in moderate electrolyte leakage across the membrane that may be reversed during recovery from LT stress (Arora and Palta, 1986). T_m_ is the midpoint temperature of this process, and can be used as an estimate of LT_50_ in MLT and more LT sensitive species or tissues, but not for those that partially or fully survive freezing stress as indicated by REL_max_ values < 0.7 (and corroborated by direct observation of injury symptoms as discussed below). The ratio T_m_/REL_max_, here called LT tolerance index (LTTI), provides a useful one-dimensional index of relative LT tolerance (Strimbeck and Schaberg, 2009). LTTI remains above -50 in needles of warm temperate and oceanic conifer species such as Sitka spruce (*Picea sitchensis*), but nears -200 in fully acclimated needles of ELT tolerant species such as Siberian spruce (*Picea obovata*) (**Figure 2**).

In conifer needles, freezing stress followed by exposure to light results in visible symptoms ranging from mild, reversible chlorosis to red–brown necrosis indicating tissue death. These can be quantified by image analysis and compared to REL measurements (Strimbeck et al., 2007). Necrosis occurs only at REL > 0.5, with complete necrosis generally occurring at REL > 0.7. In ELT tolerant species, temperatures below T_m_ or liquid nitrogen quenching from -30°C or lower result in mild to moderate chlorosis, but there is no necrosis and REL remains below 0.5 in both cases. Field observations and laboratory experiments indicate that chorosis is a reversible symptom of light x LT stress (Baronius et al., 1991; Adams, 1996) that may be exacerbated by sublethal LT stress. These observations confirm that ELT tolerant needles survive slow freezing at temperatures down to -60°C and LN_2_ quenching from temperatures below -30°C, and provide a reference scale for interpretation of REL measurements in conifer needles.

## Environmental Control Of Acclimation And Deacclimation

Seasonal changes in LT_50_ or other estimates of LT tolerance have been used to characterize acclimation and, less frequently, deacclimation under natural or controlled-environment conditions. These measurements are often used to characterize the phenology or rate of acclimation, identify environmental signals that control or affect the process, or study biochemical, gene expression, or other biological changes involved in LT tolerance. The main environmental factors that initiate and control acclimation are photoperiod in the form of increasing night length in late summer and chilling temperatures in late summer and autumn (Christersson, 1978; Bigras et al., 2001; Li et al., 2004). In some species and ecotypes, the photoperiod requirement can be bypassed by sufficient exposure to LT (Tanino et al., 2014). Deacclimation is driven mainly by warm temperatures, independent of photoperiod, resulting in increased risk of precocious deacclimation and subsequent LT injury in plants growing outside their natural range or as a result of global warming. Functional genomic and population genetic studies have identified photoperiod- and temperature-sensitive acclimation pathways under control of C-repeat binding factors (CBFs) in *Arabidopsis* (Thomashow, 1999; Lee and Thomashow, 2012), *Prunus* (Artlip et al., 2013; Wisniewski et al., 2015), and ELT tolerant *Populus* (Benedict et al., 2006; Menon et al., 2015) and *Betula* (Welling and Palva, 2008) species.

For ELT tolerant woody plants, Weiser (1970) proposed a three-stage model with the first stage initiated by decreasing photoperiod, the second stage by chilling temperatures or relatively high subfreezing temperatures, and the third stage by exposure to LTs in the -30 to -50°C range. He concluded that prolonged exposure to temperatures below -30°C is necessary for woody plants to achieve ELT tolerance. However, just a few years later Weiser coauthored a study showing that several ELT tolerant conifer and angiosperm species can attain LN_2_ quench tolerance in an artificial acclimation procedure that involved sequential storage at -3, -5, and -10°C for 14, 7, and 3 days, respectively (Sakai and Weiser, 1973). A recent comparative study of acclimation in ELT and MLT tolerant conifer species showed that the ELT tolerant species acclimated more rapidly than their MLT tolerant counterparts, and were able to survive temperatures of -40° by late October (**Figure 2**), even though temperatures remained above freezing during much of the acclimation period (Strimbeck et al., 2008). Thus, substantial acclimation can occur in ELT tolerant species in the absence of freezing temperatures, while MLT tolerant species may be more responsive to subfreezing temperatures, as suggested in a review of LT tolerance in conifers (Bigras et al., 2001).

While exposure to MLT or ILT may not be required for full acclimation, other studies (Sakai, 1966; Bigras et al., 2001; Beck et al., 2004; Søgaard et al., 2009) suggest that exposure to night frost is required for complete acclimation in ILT and ELT tolerant species, with some suggesting that a single frost event could act as a signal for further acclimation. However, some of these studies are based on field observations without a no-frost control, or, in controlled environment studies, temperature treatments may be confounded with other factors such as the duration of LT exposure or parallel changes in photoperiod. Some observed differences in response to night frost may also be due differences in the age and growing conditions of the plants used in the study, with potted seedlings in controlled environments potentially responding differently than saplings or mature trees under field conditions. There is also some indication that MLT tolerant species may be more responsive to night frost than ELT tolerant species (Bigras et al., 2001; Strimbeck and Kjellsen, 2010). A series of controlled-environment studies on potted *P. abies* plants maintained under early to mid-autumn photoperiods and temperature regimes found no effect of one or two nights at -6°C and only inconsistent effects of up to 16 frost nights or 7 days of continuous freezing as compared to unfrozen controls (Strimbeck and Kjellsen, 2010). Taken together, seasonal acclimation studies and at least some controlled environment studies indicate that acclimation in ELT species may be relatively inflexible and driven largely by short photoperiod and chilling temperatures, with only a minimal, if any, requirement for exposure to subfreezing temperature for complete acclimation.

Extreme low temperature species live in environments with severe winters, where temperatures usually remain below freezing for the entire midwinter period. These environments occur at higher latitudes where global warming has been and is generally predicted to be more extensive than at lower latitudes. This raises the possibility that winter warming may disrupt the phenology of dormancy and the acclimation–deacclimation cycle for trees and other plants in boreal and arctic regions, resulting in injury or death of exposed tissues or whole plants. Winter thaws are periods when temperatures remain above 0°C in environments where seasonal temperatures normally remain below freezing. Increases in the frequency, duration, and intensity of these events are a potential consequence of global warming, and have been observed in some locations. Winter thaws can result in precocious deacclimation, and, if followed by a return to sufficiently cold temperatures, could result in LT injury.

Studies of deacclimation in plants in general show that some species or genotypes deacclimate rapidly, while others are more or less deacclimation resistant (Kalberer et al., 2006). As an example of the former, the MLT to ILT tolerant species *P. rubens*, growing in a natural mid-elevation stand, deacclimated by as much as 14°C during a relatively extreme winter thaw (Strimbeck et al., 1995). Plants in stable environments with relatively small temperature fluctuations may be less deacclimation resistant because the first occurrence of thaw weather is a reliable signal of the arrival of spring. Some support for this hypothesis was found in a comparative study of generally MLT tolerant *Rhododendron* species (Arora and Rowland, 2011). However, the same principle could apply to ELT tolerant plants in boreal and arctic environments with stable subzero temperatures throughout the winter and where prolonged winter thaws are historically rare or non-existent. The deacclimation response could also be affected by dormancy status, which in turn may be affected by environmental temperature. Bud forcing experiments show that many woody plants transition from deep endormancy to ecodormancy after fulfillment of a chilling requirement ranging from a few weeks to a few months below some threshold temperature, usually occurring by midwinter (e.g., Rinne et al., 2001). Ecodormant tissues could be more responsive to winter thaws than fully endodormant tissues.

Little work has been done on deacclimation in ELT tolerant species. In a controlled-environment study, Ögren (2001) found a deacclimation response to thawing in *Pinus contorta* but none in high-latitude Swedish provenances of *Pinus sylvestris* and *P. abies*. Although the study does not report LT_50_s, the latter two species are typically ELT tolerant in northern and interior parts of their ranges. In seasonal monitoring of frost tolerance parameters in conifer needles, T_m_ in both MLT and ELT tolerant species groups fluctuated slightly in apparent response to winter thaw and frost periods but the ELT tolerant species maintained LN_2_ quench tolerance throughout the midwinter period (Strimbeck et al., 2008). Thus it appears that ELT tolerant species may not be completely insensitive to thaw weather, but are able to maintain nearly complete midwinter LT tolerance even in environments that are far milder than those in their natural range.

### Biochemistry of Extreme Low Temperature Tolerance

The seasonal acclimation–deacclimation cycle involves extensive changes in gene expression, biochemistry, and cellular ultrastructure (Sakai and Larcher, 1987; Li et al., 2004; Kalberer et al., 2006). These have been documented in numerous studies observing these changes during acclimation and deacclimation under natural and controlled environment conditions or, less frequently, via correlation with quantitative measures of LT tolerance. These kinds of studies have identified changes in levels of various compounds that are generally consistent during acclimation to different levels of LT tolerance and can help identify important biochemical and physicochemical process that enable cells to survive LT stress. Identification of biochemical changes that occur late in the acclimation process, during the transition from MLT to ELT tolerance, may help identify compounds and processes that are unique to ELT tolerance.

Metabolomic analysis offers a relatively new way to obtain a broad overview of biochemical processes involved in acclimation. A metabolomic study of cold and heat shock responses in *Arabidopsis* identified significant changes in 311 polar solutes in response to cold shock versus 143 responding to heat shock (Kaplan et al., 2004). Response profiles to both heat and cold were dominated by increases in numerous carbohydrates including mono-, di-, and trisaccharides, sugar alcohols, and sugar-derived organic acids. There were also increases in several protein and non-protein antioxidants, amino acids and oligopeptides. Non-polar compounds such as fatty acids were not assayed. This study provides important baseline metabolomic data for an herbaceous plant with limited LT tolerance (LT_50_ of about -11°C after cold treatment) that can be compared with early and late stages of acclimation in MLT to ELT tolerant plants.

**FIGURE 3:**
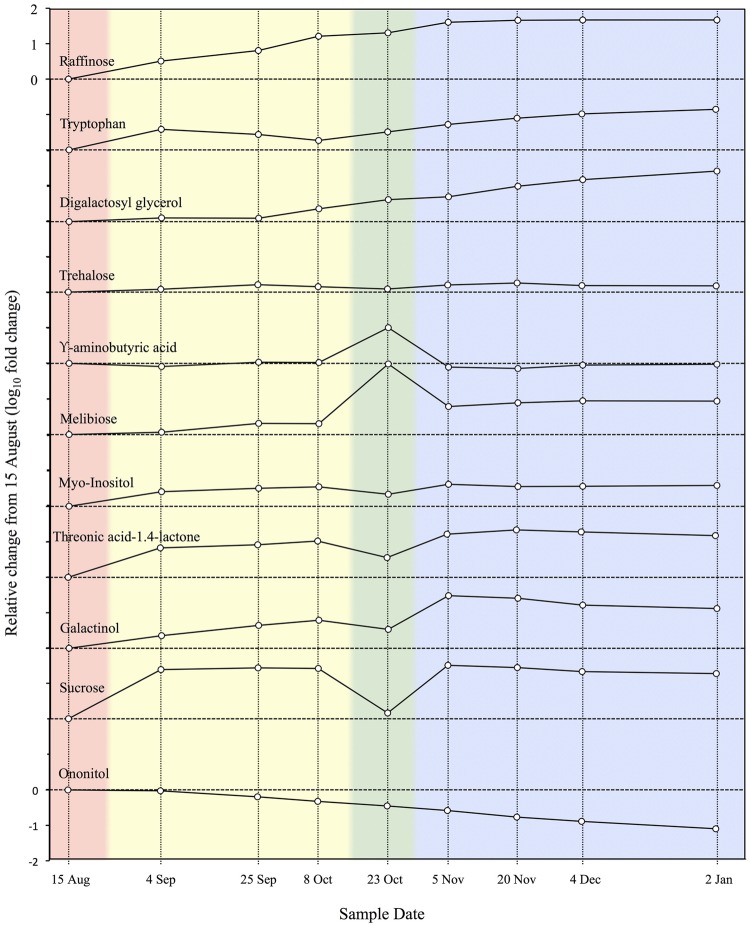
**Relative concentrations of 11 metabolites during cold acclimation in *Picea obovata*.** Colored backgrounds indicate acclimation phases determined by cluster analysis of metabolomic data: pink, pre-acclimation; yellow, early acclimation; green, late acclimation; blue, fully acclimated (adapted from Angelcheva et al., 2014).

In a metabolomic study of an ELT tolerant species, Angelcheva et al. (2014) used GC-MS to screen chloroform/methanol/water extracts from *P. obovata* needle samples collected every 2–4 weeks from late summer through midwinter. In total 223 metabolites accumulated and 52 were depleted in the overall acclimation process. A total of 68 these were identified in MS libraries, 21 of which increased during acclimation in both *P. obovata* and *Arabidopsis*, while 10 compounds showed opposite trends in the overall acclimation process. Orthogonal projections to latent structures discriminant analysis (OPLS-DA; Trygg and Wold, 2002) grouped the nine sample dates into four phases, corresponding to pre-acclimation (15 August), early acclimation (4 September – 8 October), late acclimation (23 October), and fully acclimated (5 November – 2 January) phases. These acclimation phases and the relative concentrations of 11 metabolites that changed the most over the acclimation period are shown in **Figure 3**. These results generally confirm and extend those of earlier studies showing changes in various biochemical classes during acclimation as reviewed below, and give important clues to the identity and role of compounds involved in ELT tolerance.

### Sugars

One of the most consistent changes occurring in plants during acclimation at all levels of LT tolerance is the accumulation of sugars, especially sucrose and its α-galactosyl derivatives raffinose and stachyose, usually by conversion of stored starch reserves (Sakai and Larcher, 1987). It is generally accepted that sugars have important cryoprotectant functions in LT tolerance in general, but their role in differentiating ELT tolerant from less tolerant plants and tissues is less certain.

Relatively few studies of ELT tolerant woody plants present both carbohydrate data and quantitative estimates of LT tolerance. In seasonal studies of *Robinia pseudoacacia* (Siminovitch et al., 1953) and *Morus bombycis* (Sakai, 1962a) stems, sucrose accumulated in early acclimation to about -25°C but leveled off as acclimation continued to lower temperatures, with this pattern reversed during deacclimation in the spring. Results for sucrose were similar for *Pinus strobus* needles, while raffinose was more closely associated with the acquisition and maintenance of ELT tolerance (Parker, 1959). Raffinose and stachyose but not sucrose concentrations correlated strongly with minimum survival temperatures during acclimation in *Populus tremuloides* (Cox and Stushnoff, 2001). In a study lacking direct measurement of LT tolerance, raffinose concentrations in *Cornus sericea* bark and wood increased in autumn, remained high during winter, and decreased in the spring months, while sucrose concentrations remained relatively low in winter but increased in the spring (Ashworth et al., 1993). In a 2-year study also lacking LT tolerance data, raffinose concentrations in *Pinus strobus* and *Juniperus virginiana* increased strongly and remained high during the winter months, while sucrose levels fluctuated in apparent response to environmental temperature throughout the winter. Concentrations of both sugars were considerably higher in these two northern species as compared to less LT tolerant *Pinus* and *Cupressocyparus* species (Hinesley et al., 1992). In needles of six MLT and ELT tolerant conifer species, concentrations of raffinose measured over 13 sample dates correlated strongly with T_m_ and REL_max_, with somewhat weaker correlations for stachyose (**Table 2**; Strimbeck et al., 2008). Correlations for sucrose, glucose, and fructose were weak or in the opposite direction. The inverse correlations for sucrose may be explained by its conversion to oligosaccharides during acclimation. Raffinose and stachyose comprised 25–50% of the total measured sugar in fully acclimated needles in both MLT and ELT tolerant groups. A general result that emerges from these studies is that sucrose does not have an important role in ELT tolerance, while raffinose and stachyose seem to be more important.

**Table 2:** Correlation coefficients (*r* values) between low temperature tolerance parameters and sugar contents over 13 sample dates in needles of *Abies, Picea*, and *Pinus* species in temperate and boreal groups and *Picea obovata*.

	Temperate (*n* = 117)		Boreal (*n* = 106)		*Picea obovata* only (*n* = 31)	
	T_m_	REL_max_	T_m_	REL_max_	T_m_	REL_max_
Sucrose	0.008	0.081	0.092	0.187^∗^	0.400^∗^	0.644^∗∗∗^
Glucose	0.105	-0.165	0.153	0.342^∗∗∗^	0.378^∗^	0.620^∗∗∗^
Fructose	-0.091	0.122	0.039	-0.022	0.111	0.062
Raffinose	-0.687^∗∗∗^	-0.414^∗∗∗^	-0.677^∗∗∗^	-0.819^∗∗∗^	-0.831^∗∗∗^	-0.822^∗∗∗^
Stachyose	-0.263^∗∗^	-0.151	-0.272^∗∗^	-0.377^∗∗∗^	-0.559^∗∗^	-0.548

*^∗^*p* < 0.05; ^∗∗^*p* < 0.01; ^∗∗∗^*p* < 0.001 (Strimbeck et al., 2008).*

These types of changes in carbohydrate levels were confirmed in metabolomic analysis of acclimation in *P. obovata* (**Figure 3**; Angelcheva et al., 2014). They were accompanied by a relatively minor but potentially important 1.5x increase in trehalose (Angelcheva et al., 2014), a trisaccharide that is closely linked to desiccation tolerance in animals (Crowe et al., 1984; Crowe et al., 1996). While oligosaccharide accumulation clearly plays an important role in woody plant MLT to ELT tolerance, there does not at present seem to be a unique pattern of sugar accumulation associated with ELT tolerance.

### Lipids and Fatty Acids

Like sugar accumulation, fatty acid desaturation and changes in lipid composition are broadly linked to acclimation to both chilling and freezing temperatures (Sakai and Larcher, 1987; Li et al., 2004). In ELT tolerant plants, total lipid and phospholipid content increased during acclimation in *Robinia pseudoacacia* (Siminovitch et al., 1968) and *Morus bombycis* (Yoshida, 1984), with phospholipid increases closely mirroring LT tolerance in both cases and in *Populus* sp. (Yoshida and Sakai, 1973).

In general, fatty acid composition shifts toward more unsaturated and long chain types, which are thought to help maintain membrane fluidity and prevent or lower the temperature of membrane phase changes (Uemura and Steponkus, 1999). These kinds of changes have been noted in a few ILT and ELT tolerant species, including *Populus* sp. (Yoshida and Sakai, 1973) *Morus bombycis* (Yoshida, 1984), *Picea abies* (Senser, 1982), *Pinus sylvestris* (Martz et al., 2006), *Pinus strobus* (Deyoe and Brown, 1979), and *P. obovata* (Angelcheva et al., 2014).

Changes in lipid composition are less consistent. In *Morus* plasma membranes, phosphatidylethanolamine (PE) increased and phosphatidylcholine (PC) decreased during acclimation, while in total lipids from *Populus*, both types increased, with PC showing the greater increase. During acclimation in *P. abies* thylakoid and chloroplast envelope fractions, phospholipids increased at the expense of galactolipids (Senser and Beck, 1982), but *Pinus strobus* thylakoid membranes showed opposite changes (Deyoe and Brown, 1979). Increases in phospholipids, especially PC, have been experimentally linked to membrane stability and survival during freezing of liposomes and rye protoplasts (Uemura and Steponkus, 1999; Uemura et al., 2006). Changes in lipid biochemistry clearly play an important role in LT tolerance in general, but changes unique to ELT tolerance as compared to more moderate LT tolerance are currently unclear.

### Amino Acids and Polyamines

Increases in amino acids and polyamines are another consistent response to LT and other abiotic stresses in plants (Krasensky and Jonak, 2012). These compounds are generally though to act as compatible solutes that can accumulate at high concentrations for osmotic adjustment without disrupting cell function. Various studies of ELT tolerant plants have shown increases in these compounds during acclimation. Proline and the non-protein amino acid glycine betaine are commonly associated with LT tolerance in herbaceous plants. Proline and various other amino acids increase during acclimation in woody plants, with tryptophan showing consistent increases in the ELT tolerant conifers *Picea glauca, Picea mariana, Pinus resinosa*, and *Picea obovata* (Odlum et al., 1993; Kim and Glerum, 1995; Angelcheva et al., 2014).

Ornithine and its polyamine derivatives putrescine and spermidine are often found to increase in response to stress (Krasensky and Jonak, 2012). Ornithine and putrescine increases have been observed during acclimation in *Pinus sylvestris* (Sarjala and Savonen, 1994), *Populus* sp. (Jouve et al., 1995), and *Picea obovata* (Angelcheva et al., 2014). Many of these same changes are also observed in the relatively LT sensitive *Arabidopsis* (Kaplan et al., 2004), and so are not uniquely associated with ELT tolerance.

### Proteins

In stem parenchyma cells of *Robinia pseudoacacia*, soluble protein content on a dry weight basis nearly doubles during acclimation, remains at high levels during the winter months, and decreases again during deacclimation (Siminovitch et al., 1968). This increase may involve enzymes and regulatory proteins involved in the biochemical processes described above and proteins with signaling, regulatory, protective or restorative functions for tolerating or recovering from LT stress as well as other winter stresses such as oxidative stress. Protein extracts can be screened for differential expression using 2D-gel based proteomic methods, and a subset of them can be identified or classified by various mass spectrometry methods.

Using these methods, proteomic changes during LT stress and acclimation have been explored in various tissues and cellular compartments of several herbaceous crop and model species with limited LT tolerance (Kosova et al., 2011). Hundreds of differentially accumulated protein spots have been identified in these studies, but typically, only a subset of these can be identified or classified using MS databases. Results generally indicate changes in enzymes involved in carbohydrate metabolism consistent with the sugar accumulation patterns noted above; modification of the photosynthetic system in green tissues; up-regulation of antioxidant systems; and accumulation of proteins involved in defense and stress responses. The latter group includes pathogenesis related (PR) proteins, late embryogenesis abundant (LEA) proteins, including dehydrins, that are widely associated with dehydrative stress, and heat shock proteins (HSPs) and other proteins with known or putative chaperone functions. In woody plants, proteomic changes in early acclimation have been characterized in *Populus* sp. leaves (Renaut et al., 2004) and *Prunus persica* bark (Renaut et al., 2008), with results generally similar to those for herbaceous species. A lingering challenge is to identify and characterize the many unknown proteins detected in these studies, some of which could play important roles in LT acclimation and tolerance.

A 2-D DIGE (difference in-gel electrophoresis) study of proteomic changes during acclimation in *P. obovata* found 250 differentially accumulated spots (Kjellsen et al., 2010). Of 110 proteins that showed a net accumulation during acclimation, 78 accumulated mainly in early acclimation, 28 in late acclimation, and 24 in both stages. The largest change observed for any protein during acclimation was a 17x increase, mainly in late acclimation, of a 33 kDa dehydrin. A 35 kDa non-dehydrin protein similar to an uncharacterized *P. sitchensis* protein increased by about 8x, while all other significant increases were in the 1.5–3x range, including a second 35 kDa *P. glauca*-like dehydrin that increased by about 3x. Other accumulated proteins included HSPs, AAA^+^ ATPases, a few other classes with possible roles in acclimation, and proteins associated with oxidative stress, photosynthesis, and some metabolic pathways.

Dehydrins are a subset of LEA proteins, first identified and characterized in the 1990s (Close, 1996), that are produced or accumulate in response to dehydrative stress in vascular plants, with most species producing one or more of several distinct types that vary widely in size and structure. Increases in dehydrin levels are associated with LT acclimation in numerous species, including ELT tolerant species such as *Betula pubescens* (Rinne et al., 1999), *Cornus sericea* (Sarnighausen et al., 2002), *Pinus sylvestris* (Kontunen-Soppela and Laine, 2001), *Picea glauca* (Liu et al., 2004), and *Picea obovata* (Kjellsen et al., 2013). In the latter species, 50, 34, and 32 kDa dehydrins accumulated during acclimation and dissipated during deacclimation, and immunoblotting using a more sensitive immunity-purified K-segment antibody detected three additional bands at 30, 28, and 26 kDa in fully acclimated needles. In the same study, transcripts of eight dehydrin genes increased in abundance during acclimation and decreased during deacclimation, while a ninth dehydrin followed a reverse pattern. The strong association of dehydrins with LT stress response and acclimation, as well as other kinds of stress, indicate that they have an important role in LT stress tolerance at all levels. While they fall in the same structural classes as in other species, some of the dehydrins in ELT tolerant species could have important functional characteristics that help confer ELT tolerance.

## Ultrastructure

Ultrastructural reorganization during LT acclimation has been described in needle or bark tissues of a few MLT to ELT tolerant species. In mesophyll cells of *P. abies* needles, the central vacuole is replaced by numerous small vesicles, chloroplasts and other organelles are clumped together at one end of the cell and starch granules disappear (Soikkeli, 1978). In fully acclimated *P. abies* chloroplasts, the thylakoid membranes separate and become disorganized, with few grana and numerous intermembrane plastoglobuli (Senser et al., 1975). Similar changes in chloroplast distribution and structure occur in *Abies balsamea* (Chabot and Chabot, 1975) and *Pinus sylvestris* (Martin and Oquist, 1979) needles under both natural and artificial acclimation conditions. During early acclimation in *Populus* x *canadensis* ray parenchyma cells (Sauter et al., 1996), large vacuoles present in summer disappear and protein storage vacuoles and oleosomes accumulate. Later in the process, starch stored in numerous amyloplasts disappears completely, while dense aggregations of vesicular and cisternal endoplasmic reticulum develop at the cell periphery. In *Robinia psuedoacacia* bark tissues, the plasma membrane invaginates and forms numerous small vesicles and the ER also becomes vesiculated (Pomeroy and Siminovitch, 1971). Generally similar changes occur in MLT tolerant *Prunus persica* cortical and xylem parenchyma cells (Wisniewski and Ashworth, 1986). While there are differences in the interpretation of the origin of various vesicular structures, it seems clear that LT acclimation involves massive reorganization of cellular membranes including thylakoids. Disappearance of starch granules is generally consistent with the starch to sugar conversion noted in biochemical studies, and in at least some cell types, there is an increase in protein and lipid storage structures.

## Synthesis

**FIGURE 4:**
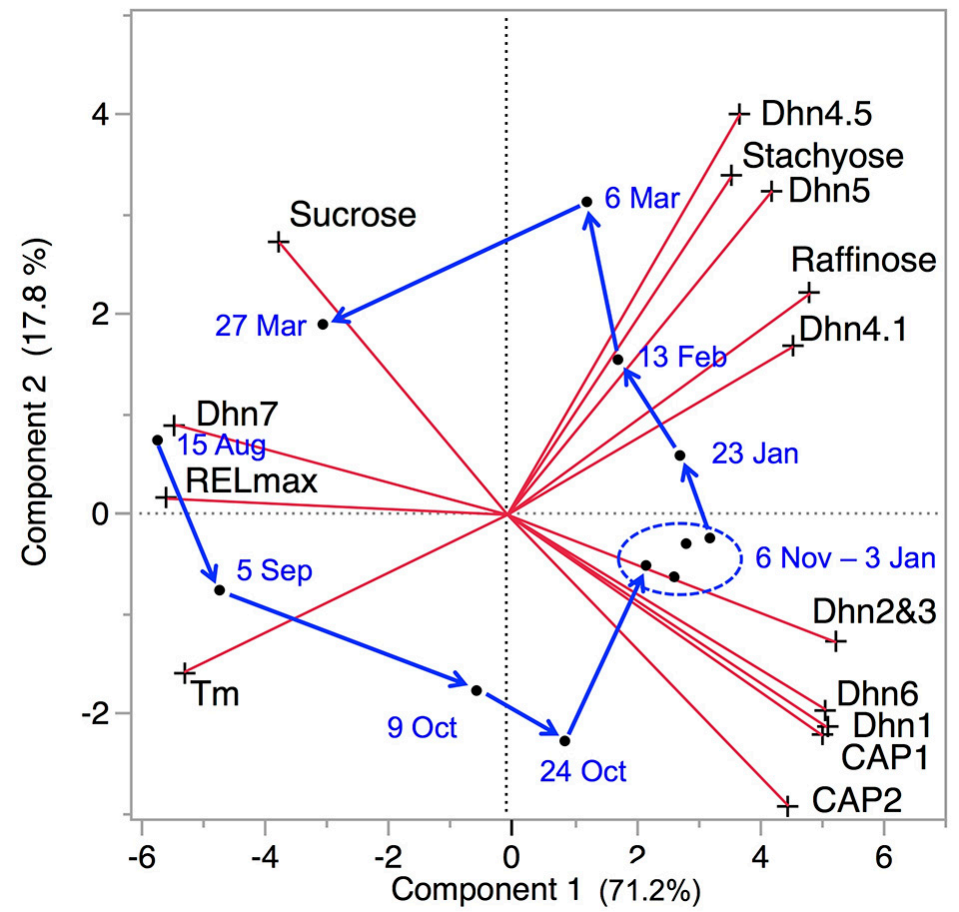
**Principle component biplot of LT tolerance, sugar, and dehydrin (Dhn and CAP) data for *P. obovata*.** Red lines indicate direction and strength of each variable. T_m_ and REL_max_ both decreased during acclimation, so greater low temperature tolerance was generally associated with higher levels of all sugars and dehydrins except sucrose and Dhn7. Dates and arrows indicate mean principle component scores for samples from three trees on each date. Data for 26 September and 24 April were excluded due to missing values for sugars and dehydrins, respectively (data from Strimbeck et al., 2008; Kjellsen et al., 2013).

A principle components analysis of LT tolerance parameters, sugar concentrations, and dehydrin transcripts measured during a complete acclimation–deacclimation cycle shows that about 90% of the variance in the total data set can be explained by the first two principle components (**Figure 4**). Raffinose, stachyose, and dehydrin transcripts all generally increase during acclimation, with a subset of dehydrins accumulating in early acclimation, while accumulation of the sugars and three other dehydrins accelerates in late acclimation. These changes are rapidly reversed during deacclimation. This overview emphasizes the importance of these two components in LT tolerance. In this section, we offer some hypotheses to explain how sugars and dehydrins function to allow plant cells and tissue to survive ELT stress.

In freezing tolerant plant and animal tissues, ice formation is extracellular and results in the dehydration of living cells as intracellular water is drawn to extracellular ice masses. Thus, freezing stress translates into dehydration stress at the cellular level (Sakai and Larcher, 1987). Freeze dehydration becomes more severe with decreasing temperature, and can result in cellular dehydration to less than 10% of original water content at temperatures of -40°C or lower, as are commonly encountered during winter in boreal regions. This basic interpretation of freezing as a dehydration stress has been understood for more than a century (reviewed in Levitt, 1980), and numerous hypotheses linking freeze dehydration stress to cell injury and death have been proposed. Xylem ray parenchyma cells and bud primordia in many temperate zone woody species avoid severe dehydration by deep supercooling, but this avoidance mechanism has a lower tolerance limit of -40 to -50°C and does not occur in ELT tolerant plants (Wisniewski et al., 2009). More recently, the focus has been on the plasma membrane as the primary site of injury (Steponkus, 1984; Wolfe and Bryant, 1999; Uemura et al., 2006).

Eukaryotic cells are packed with membranes. Reductions in cell volume caused by desiccation or freeze dehydration will inevitably force these membranes closer together. Hydration repulsion between closely appressed membranes in dehydrated cells may translate into lateral strain within the membrane with various deleterious effects (Wolfe and Bryant, 1999) that can explain the increase in REL that occurs during freezing stress. Therefore, preventing close approach of membranes or preventing membrane denaturation in partially dehydrated cells may be of primary importance in surviving desiccation and freeze dehydration stress. Oligosaccharide accumulation, changes membrane lipid composition, and dehydrins all have potential roles in this strategy.

Relative electrolyte leakage-temperature response curves (**Figure 1**) show that an increase in electrolyte leakage across the plasma membrane is a basic and measurable response to LT or freeze-dehydration stress. The LT tolerance parameters T_m_ and REL_max_ offer two different ways to quantify this response. T_m_ is an estimate of the midpoint temperature of the response to freezing stress, regardless of whether the tissue survives or not. REL_max_ represents the amplitude of increased leakage that can be achieved by slow freezing, with values >0.5 generally indicating partial to complete tissue death.

One likely explanation for the sigmoid shift in REL centered on T_m_ is a liquid crystal to gel phase transition or more drastic reorganization of membranes in response to some combination of LT and dehydration (Steponkus, 1984; Williams and Quinn, 1987; Wolfe and Bryant, 1999). This change in membrane structure results in membrane leakage after thawing. At temperatures below LT_50_ in MLT tolerant species, these kinds of transitions are irreversible, so that the cell is unable to regain osmotic control after thawing and eventually dies. However, in ELT tolerant species, where freezing stress results in only moderate increases in REL, there may be either no significant reorganization of the membrane at T_m_ or whatever reorganization that does occur may be reversible given sufficient recovery time, as suggested by restoration of semipermeability following sublethal stress in onion bulb cells (Arora and Palta, 1986). The shift in T_m_ toward lower temperatures that occurs during acclimation in all species may be a result of changes in membrane composition that allow the plasma membrane to maintain stability at lower temperatures and greater levels of dehydration. Numerous studies have shown that fatty acid desaturation and changes in membrane lipid composition occur during acclimation (Sakai and Larcher, 1987). These changes in membrane composition can lower phase change temperatures and affect other membrane behaviors under freezing stress (Uemura et al., 2006). While the difference between MLT and ELT tolerance could be partially explained by these kinds of differences in membrane composition, in ELT tolerant species the shift in T_m_ seems to reach an acclimation limit at about -50°C (**Figure 1**), but these species are able to completely survive much lower temperatures. This indicates that ELT tolerant species have other mechanisms for surviving extreme freezing stress.

The signature transition to LN_2_ quench tolerance that occurs during slow cooling between -20 and -30°C in ELT tolerant species can be explained by cytoplasmic vitrification, the transition from a fluid to an amorphous solid or glassy state. Vitrification is thought to be an important mechanism of desiccation tolerance in seeds and some other desiccation tolerant plant tissues, and may also occur as a result of freeze dehydration (Koster, 1991; Buitink and Leprince, 2004). The glassy state is, in effect, a kind of molecular suspended animation in which molecular movement, including further dehydration and deleterious chemical reactions, is effectively arrested at all lower temperatures.

Although cells at -20 to -30°C are already substantially dehydrated, vitrification of intermembrane cytoplasm in this temperature range would prevent any further dehydration, which would in turn prevent close approach of membranes and associated lesions. Cytoplasmic vitrification can dramatically affect membrane stability under dehydration stress. In model sucrose-water-lipid systems, vitrification in the sugar-water phase decreases the temperature of the liquid crystal to gel phase transition in the lipid phase by up to 57°C (Koster et al., 2000). This leads to the specific hypothesis that membrane damage will be prevented if vitrification occurs at a temperature above T_m_.

Evidence of vitrification has been reported in frozen plant tissues (Hirsh, 1987; Vertucci and Stushnoff, 1992). Glass transitions can be detected by differential scanning calorimetry (DSC), electron spin resonance, or nuclear magnetic resonance methods as a step change in heat capacity or other measures of molecular mobility. Using modulated temperature DSC, a weak glass transition has been detected in a few *Picea* needle samples at around -22°C, right in the range where tissues acquire LN_2_ quench tolerance (Strimbeck and Schaberg, 2009), but not in most samples under similar or a variety of other experimental conditions. Glass transitions in frozen plant tissues may be difficult to detect for two reasons. First, the relatively weak change in heat capacity of the dehydrated cytoplasm may be diluted by the presence of large amounts of extracellular ice. Second, the change in heat capacity may occur over a much broader temperature range in complex mixtures, such as the cytoplasm, as compared to sucrose-water and other simple systems.

Freeze-concentrated sucrose solutions vitrify readily, with glass transitions at about -41°C (Goff et al., 2002). The glass transition temperature is somewhat higher in sucrose-raffinose-water mixtures, but pure raffinose solutions tend to undergo eutectic crystallization. In *P. obovata*, raffinose and stachyose were closely related to LT tolerance (**Table 1**, **Figure 4**), highlighting the likely roles of these sugars in ELT tolerance. The increase in trehalose observed in *P. obovata* (Angelcheva et al., 2014) may also be significant, as this disaccharide vitrifies at high temperatures and is strongly associated with desiccation tolerance in animals (Crowe et al., 1998; Wolfe and Bryant, 1999). While sugars may have other protective effects, the well-documented vitrification behavior of sugar solutions indicates that vitrification is likely, if not inevitable, during freeze-dehydration in the sugar-enriched cytoplasm of LT acclimated cells.

Glass transition behavior and temperature could also be affected by other cytoplasmic components, especially unstructured polymers, which are hypothesized to vitrify via “molecular entanglement” of polymer chains (Levine and Slade, 1992). This observation suggests a specific role for dehydrins in ELT tolerance. All dehydrins contain one or more copies of the K segment, a highly conserved 15 amino acid segment with the consensus sequence EKKGIMDKIKEKLPG. Some classes of dehydrins contain from one to several copies of the seven-residue Y segment ((V/T)D(E/Q)YGNP) and an S-segment with as many as nine consecutive serine residues. Outside of these conserved segments, dehydrins are highly hydrophilic and disordered with little recognizable sequence conservation. Dehydrins have been proposed or shown to have antifreeze, metal-binding, antioxidant, protein binding, or membrane binding properties (Rorat, 2006; Eriksson and Harryson, 2011). The K segment forms an amphipathic α-helix in non-polar environments (Ismail et al., 1999) that binds to lipid vesicles (Koag et al., 2003; Koag et al., 2009; Eriksson et al., 2011). Membrane binding is likely a key property of dehydrins, suggesting a role in protecting membranes against dehydration stress. Macromolecules may be excluded from intermembrane spaces and therefore have no cryoprotective effect (Wolfe and Bryant, 1999). We propose that the K segment anchors dehydrins to membranes, with the unstructured regions of the protein free to interact with sugars to promote intermembrane vitrification via molecular entanglement.

While this discussion focuses on vitrification, complete vitrification may not be an absolute requirement. An increase in viscosity to an intermediate plastic or rubbery state could slow molecular motion enough to stabilize cells for weeks or months (Wolfe et al., 2002). Furthermore, even in the absence of vitrification, the unstructured regions of dehydrins could act stearically as “molecular spacers,” preventing the close approach of membranes and diminishing the strains in membranes caused by repulsive forces. The different types of dehydrins found in most plant species (i.e., ten in the *Arabidopsis* genome and at least nine in *P. obovata*), may be targeted to specific membranes or cell compartments so that all membranes receive sufficient protection.

Extreme low temperature tolerance is an intriguing phenomenon with potentially high relevance for the development or improvement of technologies for dried and frozen preservation of drugs, foods, cells, tissues, and perhaps even organs or whole organisms. Beginning with the pioneering work of Sakai (1960, 1962a, 1965), the LT tolerance characteristics and geographic distribution of ELT tolerant woody species have been defined. It seems clear that no one metabolite or protein is responsible for the ability of plant tissues to survive at temperatures approaching absolute zero. With the introduction of screening technologies such as proteomics and metabolomics, backed up by decades of work on biochemical changes during acclimation, many of the major molecular actors have been identified. *In vitro* and *in vivo* functional analyses of these components, separately and in combination, should help to complete the picture.

## Conflict of Interest Statement

The authors declare that the research was conducted in the absence of any commercial or financial relationships that could be construed as a potential conflict of interest.
